# Comparison of Bipolar Plasma Vaporization versus Standard Holmium Laser Enucleation of the Prostate: Surgical Procedures and Clinical Outcomes for Small Prostate Volumes

**DOI:** 10.3390/jcm8071007

**Published:** 2019-07-10

**Authors:** Kang Sup Kim, Sung Hyun Lee, Hyuk Jin Cho, Hong Jin Suh, Dong Hwan Lee, Yong Sun Choi

**Affiliations:** 1 Department of Urology, Incheon St. Mary’s Hospital, College of Medicine, The Catholic University of Korea, 59 Dongsu-ro, Bupyeong-gu, Incheon 2143, Korea; 2 Department of Anesthesiology and Pain Medicine, Kangbuk Samsung Hospital, Sungkyunkwan University School of Medicine, 29, Saemunan-ro, Jongno-gu, Seoul 03181, Korea; 3 Department of Urology, Seoul St. Mary’s Hospital, College of Medicine, The Catholic University of Korea, 222, Banpo-Daero, Seocho-Gu, Seoul 137-701, Korea; 4 Department of Urology, Eunpyeong St. Mary’s Hospital, College of Medicine, The Catholic University of Korea, 1021, Tongil-ro, Eunpyeong-gu, Seoul 03312, Korea

**Keywords:** bipolar plasma vaporization, holmium laser enucleation, lower urinary tract symptoms

## Abstract

Bipolar plasma vaporization of the prostate (BPVP) is an attractive alternative to resection. There are numerous studies comparing transurethral resection of prostate or photoselective vaporization of the prostate with BPVP; however, there is a lack of data comparing holmium laser enucleation of the prostate (HoLEP) with BPVP. We aimed to compare HoLEP and BPVP with a focus on functional outcomes, safety, and complications. Methods: From January 2017 to June 2018, patients who underwent HoLEP or BPVP for benign prostatic hypertrophy were enrolled in this study. Inclusion criteria were a prostate volume <40 mL measured by transrectal ultrasound, international prostate symptom score (IPSS) >7, maximum urinary flow rate (Qmax) <15 mL/s, and postvoiding residual volume (PVR) >100 mL. Perioperative and postoperative parameters including IPSS, Qmax, quality of life, PVR, and complications were compared between groups. Results: Sixty-three patients were enrolled in this study. There were small differences in perioperative parameters. Hospital stays and catheterization periods were significantly shorter in the BPVP group. The postoperative complications were comparable between groups. PVR was comparable in both groups except for 1 month postoperatively. The incontinence rate was higher in the HoLEP group, but without statistical significance. Conclusion: In terms of surgical safety and efficacy as well as patient comfort, BPVP is comparable with HoLEP for small prostate volumes. BPVP can be a viable alternative technique in small BPH surgical treatment.

## 1. Introduction

Lower urinary tract symptoms (LUTS) are common complaints in adult men, and cause a major impact on quality of life (QoL) and impose a substantial economic burden [1]. The first-line treatment for symptomatic LUTS with benign prostate hyperplasia (BPH) is medical treatment, and there are several indications for surgery. Transurethral resection of the prostate (TURP) and open simple prostatectomy have been the gold standards for surgical treatment for BPH for more than nine decades [2,3]. However, holmium laser enucleation of the prostate (HoLEP) is gaining acceptance as the new gold standard surgery for BPH and LUTS [4]. HoLEP has been shown to be superior to open simple prostatectomy in terms of length of hospital stays, transfusion rates, and catheterization times [5]. Despite these clear advantages of HoLEP, the steep learning curve, inability to easily convert to conventional resection, and added expenses of the morcellation device and high-energy holmium laser are obstacles to widespread adoption of HoLEP [6].

Various new surgical techniques such as photo vaporization of prostate, transurethral enucleation of prostate, aqua ablation, and water vaporizations were introduced as an alternative to these shortcomings of HoLEP technique. But those techniques require special equipment, have a steep learning curve, and the cost is expensive. Furthermore, there has been a demand for similar, yet better techniques for conventional TURP. Among these various techniques, bipolar plasma vaporization of prostate (BPVP) technique has been introduced as a reasonable alternative for TURP. BPVP does not require a steep learning curve, a laser generator, or special equipment other than a regular resectoscope. BPVP also provides real-time tissue ablation with excellent hemostasis, even in high-risk patients, and has gained increasing acceptance [7].

There are numerous studies comparing both PVP and BPVP; however, there is a lack of data comparing the HoLEP technique with BPVP to determine the superior modality. Therefore, we performed a retrospective comparative study between HoLEP and BPVP with a focus on the functional outcomes, safety, and complications for both procedures.

## 2. Patients and Methods

### 2.1. Ethics Statement

The study protocol was approved by the Institutional Review Board and Medical Ethics Committee of the Catholic University of Korea (approval number: OC17RESI0150). All research was conducted in accordance with the Declaration of Helsinki.

### 2.2. Study Enrollment

After the local ethics approval, the patients’ medical record who underwent either HoLEP or BPVP between January 2017 to June 2018 were reviewed retrospectively. All patient’s records were anonymized and de-identified prior to analysis. A standard evaluation protocol including general clinical evaluation, digital rectal examination (DRE), complete blood count, blood chemistries, prostate specific antigen (PSA), urinalysis, urine culture, IPSS, QoL, uroflowmetry, transrectal ultrasonography to measure prostate volume, and post voiding residual volume were reviewed. 

Inclusion criteria were a prostate volume < 40 mL measured by transrectal ultrasound (TRUS), international prostate symptom score > 7, maximum urinary flow rate (Qmax) < 15 mL/s, postvoiding residual volume > 100 mL, and recurrent or persistent gross hematuria due to enlarged prostate or bladder calculi. Prostate volume was calculated using a conventional formula (width × length × height × π/6). Patients who had prostate or bladder carcinoma, permanent anticoagulation therapy, neurogenic bladder, and previous urethral or prostate surgery were excluded. If the patient had a PSA level > 4.0 ng/mL, abnormal DRE, or hypoechogenic lesion on TRUS, then sextant TRUS-guided prostate biopsy was done to exclude malignancy.

### 2.3. Study Endpoints

The primary endpoints were the efficacy of BPVP vs. that of standard HoLEP as measured by changes in IPSS, quality of life, Qmax, and residual urine volume. The secondary endpoints were safety of BPVP vs. HoLEP as assessed by hemoglobin change during surgery, and operation times, as well as postoperative complications, including dysuria, uncontrolled hematuria requiring clot evacuation, re-operation or transfusion, re-catheterization, febrile urinary tract infection (UTI), and incontinence.

### 2.4. Surgical Intervention

HoLEP and BPVP were done by two experienced urologists, respectively. Two experienced urologists performed more than 100 HoLEP and BPVP before this study, respectively. We consider that two experienced urologists overcame the learning curve of these surgical technique. HoLEP was performed using a 120 W Holmium:YAG laser (VersaPulse PowerSuite, Lumenis Surgical, San Jose, CA, USA) with a 550-nm end-firing fiber (SlimLine, Lumenis). A 26-Fr continuous-flow resectoscope with saline irrigation was used. The laser settings were 2.5 J and 40 Hz. After enucleation of the adenoma and control of bleeding, enucleated adenomas were removed from the bladder using a mechanical tissue morcellator (Versa-Cut, Lumenis) with an indirect nephroscope. [8] The BPVP technique required the Olympus SurgMaster UES-40 bipolar generator (Olympus, Tokyo, Japan) under continuous flow saline irrigation with a standard button- or mushroom-type vapo-resection electrode. During BPVP, the button-type electrode presenting a plasma corona on its surface was moved forward and backward in close contact with the prostatic tissue, which was vaporized layer-by-layer until reaching the surgical prostate capsule. The BPVP output was controlled flexibly to achieve a bloodless operation field for proper tissue vaporization and simultaneous hemostasis.

### 2.5. Statistical Analysis

The Pearson chi-square test was used to compare the categorical variables. The student’s *t*-test was used to analyze two continuous variables. A *p*-value < 0.05 was considered statistically significant. All statistical analyses were conducted using SPSS version 19.0 (IBM Inc. Armonk, NY, USA).

## 3. Results

From January 2017 to June 2018, a total of 63 patients who underwent HoLEP or BPVP with BPH and severe LUTS were enrolled.

Preoperative values were determined for each group in terms of general demographics, mean IPSS, QoL, Qmax, postvoiding residual volume, hemoglobin, PSA, and prostate volume. There were no statistically significant differences between them (Table 1).

During the surgery, the BPVP and HoLEP were successfully performed in almost all cases. Intraoperative data including the operation time, blood losses were comparable in both groups. The incidence of capsular perforation was higher in the HoLEP group (3.2% vs. 12.5%); however, it was not significantly different. (*p* = 0.173) Length of hospital stay and catheterization times were significantly shorter in the BPVP group than in the HoLEP group (*p* = 0.001, *p* = 0.001, respectively; Table 2).

Postoperative complications were gross hematuria requiring coagulation or blood transfusion and febrile UTI. Those complications were analyzed using the modified Clavien Classification. There was no significant difference between groups (Table 3).

During the 6-month follow-up period, IPSS, IPSS voiding, IPSS storage, and QoL data were collected and analyzed (Table 4). The IPSS, IPSS voiding, IPSS storage, and QoL, Qmax and residual volume measurements were done at discharge, postoperative 1, 3, and 6 months. Compared with preoperative data, there were significant serial sequential reductions in IPSS and QoL scores in both groups. However, significant differences between groups were not noted.

**Figure 1:**
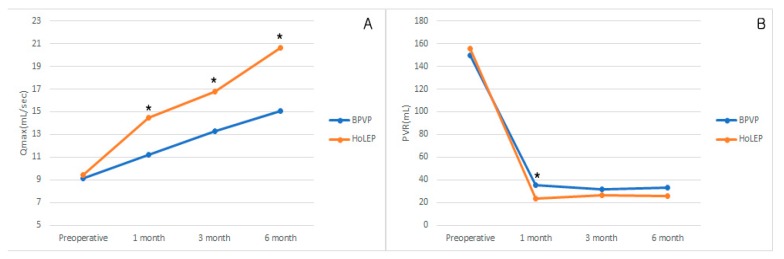
6-month follow-up data of IPSS, IPSS voiding, IPSS storage, and QoL. (**A**) Qmax and PVR follow-up results of BPVP and HoLEP (**B**), BPVP = bipolar plasma vaporization of prostate, HoLEP = holmium laser enucleation of the prostate, IPSS = international prostate symptom score, Qmax = maximum urinary flow rate, PVR = postvoiding residual volume, * *p* < 0.05; compared BPVP with HoLEP.

The Qmax and RU F/U results were significantly different from one another. There was serial sequential improvement of Qmax and there were significant differences between groups. (Figure 1A). The PVR improved after the surgery; however, there were no significant differences between groups except for 1 month postoperatively (35.2 mL vs. 28.8 mL, *p* = 0.037) (Figure 1B).

**Figure 2:**
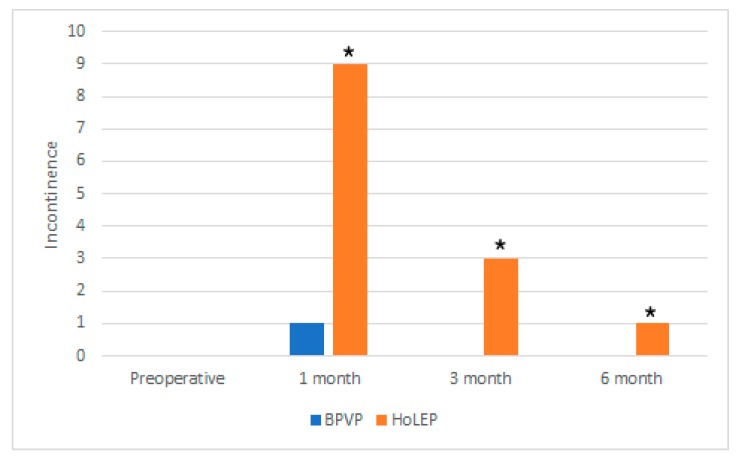
The number of patients reporting incontinence after BPVP and HoLEP for 6 months. BPVP = bipolar plasma vaporization of prostate, HoLEP = holmium laser enucleation of the prostate, * *p* < 0.05; compared BPVP with HoLEP.

The number of patients reporting incontinence was nine at 1 month post-HoLEP, three at 3 months, and one at 6 months. Compared with the BPVP group, there was a definite difference between groups; however, the difference decreased over time (Figure 2).

## 4. Discussion

The first-line treatment for symptomatic LUTS with benign prostate hyperplasia (BPH) is considered medical treatment, and there are several indications for surgery [3]. Prostate volume is a critical factor for surgical management decision. Open simple prostatectomy and TURP are the standard procedures for moderate or large prostates, and several alternative techniques including HoLEP and BPVP have been introduced to overcome the drawbacks of standard procedure. However, for patients who fail medical therapy with small prostates, urologists might hesitate to recommend surgery because of the possibility of decreased efficacy.

Based on previous studies, prostate size < 40 mL is the rational cut off level for small prostate volume [9,10]. Kaplan analyzed cohorts with prostate volumes >40 mL, 25–40 mL, and <25 mL and found a benefit from combination therapy with α-adrenergic blocker and 5-α reductase inhibitors compared with patients with prostate volumes <25 mL [11]. Therefore, prostate size is related to prognosis in BPH.

BPVP is an alternative to TURP. The equipment and surgical techniques are similar to those of the traditional TURP technique. Therefore, BPVP does not require a steep learning curve. Surgeons can easily adopt the new technique without specialized equipment. Compared with conventional TURP, BPVP technique has superior efficacy and satisfactory reasonable complication rate, BPVP can be a valuable endoscopic treatment alternative for BPH patients [12,13].

Several randomized studies comparing bipolar TURP, monopolar TURP with plasma vaporization concluded the positive findings for the efficacy and safety of bipolar plasma vaporization for long time [12,14,15,16].

In our experience, BPVP provides a remarkable intraoperative visual field by reducing bleeding and detailed visual differentiation of the adenoma tissue from the surgical prostate capsule. The final inspection of the prostate shows the large vaporized prostate fossa with smooth surface and sharp edge of the vaporized area without irregular or adenoma tissues emerging. The advantage of the excellent operative field may explain the lower mean hemoglobin changes and lower capsular perforation rates in the BPVP group. Though the difference was not significant, the hemoglobin changes and capsular perforation rates were relatively small in the BPVP group. Furthermore, the length of hospital stays and catheterization periods in the BPVP group were significantly shorter than in the HoLEP group. These perioperative results were consistent with those of another previous bipolar surgical study [13].

Postoperative complications were comparable between groups. Even though the statistical differences were not significant, hematuria and hematuria-related complications including clot retention, blood transfusion, and bleeding requiring surgery were in high in the HoLEP group; nevertheless, these complications are critical in terms of patient care. Other studies reported significant differences in rates of these complications compared with those of bipolar surgery [17,18,19]. Further large-population and prospective studies are needed to confirm the lower postoperative complication rates following BPVP. In addition to these complications, dysuria can be the most bothersome postoperative complication of BPVP. The early irritative symptoms after bipolar vaporization varied from 2% to 13% and remain a subject of debate [18,20,21]. Our dysuria rate was 12.9%. This was comparable to that of the HoLEP group. Larger sample size with long term follow up period should be carried for more precise safety result.

Postoperative parameters including IPSS, QoL, Qmax, and PVR showed clear sequential improvements in both groups. The improvement of IPSS and QoL were comparable in both groups. The Qmax improvements were significant and more prominent in the HoLEP group. The Qmax is important for BPH with LUTS patients, because Qmax < 10 mL/s presents higher risks of urinary retention and subsequent prostate surgery [22]. Despite the fact that the BPVP group showed significantly lower Qmax than did the HoLEP group, the mean postoperative Qmax was over 10 mL/s, and increased over time. The immediate postoperative Qmax was higher in HoLEP group than BPVP group, but the difference converge with time. But, in HoLEP group, the incontinence was higher in postoperative and last longer than BPVP group. We assume this is because the HoLEP group achieved more radical tissue enucleation than did the BPVP group. However, these results converged with time. Nevertheless, IPSS and PVR remained comparable and showed sequential improvements in each group throughout the evaluation period.

Urinary incontinence is a common major complication of HoLEP. The number of patients with postoperative incontinence in the HoLEP group was high and decreased gradually. However, patient emotional stress during the incontinence period is high enough for them to complain about their problems to surgeon. In the BPVP group, the incontinence rate was much lower than in the HoLEP group, and this low incontinence rate can be considered another advantage of BPVP. We believe that the radical tissue enucleation achieves better Qmax, however, at the expense of a higher incontinence rate.

One limitation of this study was the relatively small prostate size. The mean prostate size of this study was about 40 g, not large prostates. Despite these prostate sizes, the patients presented severe LUTS and complaints decreased after surgery. Another limitation is the small population and selection bias. Because this was not a prospective, randomized study, patient enrollment was not randomized and large enough to draw a generalizable conclusion.

Despite these limitations, the result suggests the efficacy and safety of BPVP. To our knowledge, this study is the first study to compare BPVP and HoLEP.

## 5. Conclusions

In terms of surgical safety, efficacy, and patient comfort, BPVP is comparable to HoLEP for small prostate volumes. BPVP is a viable alternative for surgical treatment of small BPH.

## Figures and Tables

**Table 1:** Preoperative data of bipolar plasma vaporization of the prostate (BPVP) and holmium laser enucleation of the prostate (HoLEP) group.

	BPVP (*n* = 31)	HoLEP (*n* = 32)	*p*-Value
Age	69.81 ± 7.54	70.23 ± 5.61	0.803
Prostate size (g)	37.04 ± 29.41	36.01 ± 25.78	0.481
Transitional zone volume (mL)	12.3 ± 15.37	13.62 ± 18.22	0.497
PSA (ng/mL)	2.11 ± 6.38	2.18 ± 6.13	0.965
Total IPSS	20.7 ± 8.1	21.8 ± 8.5	0.241
Voiding IPSS	12.7 ± 4.9	14.3 ± 5.7	0.057
Storage IPSS	8.5 ± 4.4	9.0 ± 5.3	0.941
IPSS QoL	4.5 ± 2.8	5.1 ± 1.8	0.641
Qmax (mL/s)	9.09 ± 2.37	9.45 ± 3.59	0.447
PVR (mL)	149.8 ± 68.1	155.7 ± 104.9	0.119

BPVP = bipolar plasma vaporization of prostate, HoLEP = holmium laser enucleation of the prostate, IPSS = international prostate symptom score, Qmax = maximum urinary flow rate, PVR = postvoiding residual volume.

**Table 2:** Perioperative and postoperative results of BPVP and HoLEP group.

	BPVP (*n* = 31)	HoLEP (*n* = 32)	*p*-Value
Operation time (min)	69.81 ± 7.54	70.23 ± 5.61	0.803
Hemoglobin change	47.04 ± 29.41	52.01 ± 25.78	0.481
Vaporization/Enucleation time (min)	22.3 ± 15.37	28.62 ± 18.22	0.497
Morcellation time (min)		3.18 ± 6.13	
Enucleation weight (g)		11.8 ± 8.5	
Capsular perforation (case)	1 (3.2%)	4 (12.5%)	0.173
Hospital stay (day)	2.1 ± 1.9	4.1 ± 1.3	0.001 *
Catheterization period (h)	25.7 ± 2.1	51.1 ± 1.4	0.001 *

*: Compared BPVP with HoLEP.

**Table 3:** Postoperative complications of BPVP and HoLEP group with modified Clavien Classification.

	BPVP (*n* = 31)	HoLEP (*n* = 32)	*p*-Value
Grade 1			
Clot retention	0	4	0.113
Mild to moderate dysuria	4	2	0.164
Re-catheterization	1	3	0.317
Grade 2			
Blood transfusion	0	2	0.157
Febrile UTI	1	1	0.982
Grade 3			
Bleeding requiring surgery	0	2	0.157

**Table 4:** Postoperative 6-month IPSS, IPSS voiding, IPSS storage, and QoL data.

		Preoperative	Postoperative		
			1 month	3 months	6 months
IPSS total	BPVP	20.7 ± 8.1	11.6 ± 8.3	9.8 ± 4.1	8.1 ± 3.5
	HoLEP	21.8 ± 8.5	11.5 ± 7.2	9.3 ± 5.1	8.4 ± 5.4
	*p*-value	0.241	0.098	0.184	0.217
IPSS voiding	BPVP	12.7 ± 4.9	4.6 ± 4.3	4.0 ± 4.1	3.8 ± 3.5
	HoLEP	14.3 ± 5.7	4.8 ± 4.9	3.8 ± 3.2	3.5 ± 3.3
	*p*-value	0.057	0.083	0.126	0.159
IPSS storage	BPVP	8.5 ± 4.4	6.4 ± 3.1	5.8 ± 4.1	4.1 ± 3.5
	HoLEP	9.0 ± 5.3	6.6 ± 3.2	5.7 ± 4.4	4.3 ± 3.4
	*p*-value	0.941	0.541	0.499	0.287
QoL score	BPVP	4.5 ± 2.8	2.6 ± 1.7	2.3 ± 1.6	2.1 ± 1.5
	HoLEP	5.1 ± 1.8	2.7 ± 1.4	2.4 ± 1.4	2.3 ± 1.5
	*p*-value	0.641	0.241	0.497	0.481

IPSS = international prostate symptom score, QoL = quality of life, BPVP = bipolar plasma vaporization of prostate, HoLEP = holmium laser enucleation of the prostate.
